# Dopamine, uncertainty and TD learning

**DOI:** 10.1186/1744-9081-1-6

**Published:** 2005-05-04

**Authors:** Yael Niv, Michael O Duff, Peter Dayan

**Affiliations:** 1 Interdisciplinary Center for Neural Computation, Hebrew University, Jerusalem, Israel; 2 Gatsby Computational Neuroscience Unit, University College London, London, UK

## Abstract

Substantial evidence suggests that the phasic activities of dopaminergic neurons in the primate midbrain represent a temporal difference (TD) error in predictions of future reward, with increases above and decreases below baseline consequent on positive and negative prediction errors, respectively. However, dopamine cells have very low baseline activity, which implies that the representation of these two sorts of error is asymmetric. We explore the implications of this seemingly innocuous asymmetry for the interpretation of dopaminergic firing patterns in experiments with probabilistic rewards which bring about persistent prediction errors. In particular, we show that when averaging the non-stationary prediction errors across trials, a ramping in the activity of the dopamine neurons should be apparent, whose magnitude is dependent on the learning rate. This exact phenomenon was observed in a recent experiment, though being interpreted there in antipodal terms as a within-trial encoding of uncertainty.

## Introduction

There is an impressively large body of physiological, imaging, and psychopharmacological data regarding the phasic activity of dopaminergic (DA) cells in the midbrains of monkeys, rats and humans in classical and instrumental conditioning tasks involving predictions of future rewards [1-5]. These data have been taken to suggest [6,7] that the activity of DA neurons represents temporal difference (TD) errors in the predictions of future reward [8,9]. This TD theory of dopamine provides a precise computational foundation for understanding a host of behavioural and neural data. Furthermore, it suggests that DA provides a signal that is theoretically appropriate for controlling learning of both predictions and reward-optimising actions.

Some of the most compelling evidence in favour of the TD theory comes from studies investigating the phasic activation of dopamine cells in response to arbitrary stimuli (such as fractal patterns on a monitor) that predict the proximate availability of rewards (such as drops of juice). In many variants, these have shown that with training, phasic DA signals transfer from the time of the initially unpredictable reward, to the time of the earliest cue predicting a reward. This is exactly the expected outcome for a temporal-difference based prediction error (*eg. *[1,2,10-13]). The basic finding [7] is that when a reward is unexpected (which is inevitable in early trials), dopamine cells respond strongly to it. When a reward is predicted, however, the cells respond to the predictor, and not to the now-expected reward.

If a predicted reward is unexpectedly omitted, then the cells are phasically inhibited at the normal time of the reward, an inhibition which reveals the precise timing of the reward prediction [10], and whose temporal metrics are currently under a forensic spotlight [14]. The shift in activity from the time of reward to the time of the predictor resembles the shift of the animal's appetitive behavioural reaction from the time of the reward (the unconditioned stimulus) to that of the conditioned stimulus in classical conditioning experiments [7,10].

In a most interesting recent study, Fiorillo *et al. *[15] examined the case of partial reinforcement, in which there is persistent, ineluctable, prediction error on every single trial. A straightforward interpretation of the TD prediction error hypothesis would suggest that in this case (a) dopamine activity at the time of the predictive stimuli would scale with the probability of reward, and (b) on average over trials, the dopaminergic response after the stimulus and all the way to the time of the reward, should be zero. Although the first hypothesis was confirmed in the experiments, the second was not. The between-trial averaged responses showed a clear ramping of activity during the delay between stimulus onset and reward that seemed inconsistent with the TD account. Fiorillo *et al. *hypothesised that this activity represents the uncertainty in reward delivery, rather than a prediction error.

In this paper, we visit the issue of persistent prediction error. We show that a crucial asymmetry in the coding of positive and negative prediction errors leads one to expect the ramping in the between-trial average dopamine signal, and also accounts well for two further features of the DA signal – apparent persistent activity at the time of the (potential) reward, and disappearance (or at least weakening) of the ramping signal, but not the signal at the time of reward, in the face of trace rather than delay conditioning. Both of these phenomena have also been observed in the related instrumental conditioning experiments of Morris *et al. *[16]. Finally, we interpret the ramping signal as the best evidence available at present for the nature of the learning mechanism by which the shift in dopamine activity to the time of the predictive stimuli occurs.

## Uncertainty in reward occurrence: DA ramping

Fiorillo *et al. *[15] associated the presentation of five different visual stimuli to macaques with the delayed, *probabilistic *(*p*_*r *_= 0, 0.25, 0.5, 0.75, 1) delivery of juice rewards. They used a delay conditioning paradigm, in which the stimulus persists for a fixed interval of 2s, with reward being delivered when the stimulus disappears. After training, the monkeys' anticipatory licking behavior indicated that they were aware of the different reward probabilities associated with each stimulus.

**Figure 1:**
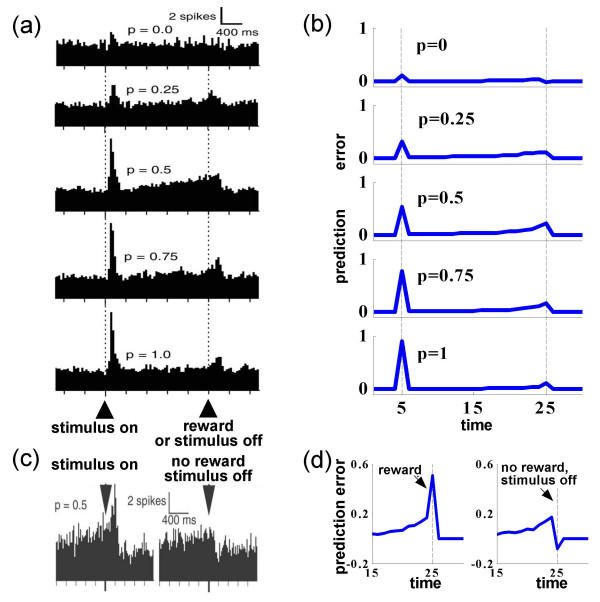
**Averaged prediction errors in a probabilistic reward task **(a) DA response in trials with different reward probabilities. Population peri-stimulus time histograms (PSTHs) show the summed spiking activity of several DA neurons over many trials, for each *p*_*r*_, pooled over rewarded and unrewarded trials at intermediate probabilities. (b) TD prediction error with asymmetric scaling. In the simulated task, in each trial one of five stimuli was randomly chosen and displayed at time *t *= 5. The stimulus was turned off at *t *= 25, at which time a reward was given with a probability of *p*_*r *_specified by the stimulus. We used a tapped delay-line representation of the stimuli (see text), with each stimulus represented by a different set of units ('neurons'). The TD error was *δ*(*t*) = *r*(*t*) + **w**(*t *- 1)·**x**(*t*) - **w**(*t *- 1)·**x**(*t *- 1), with *r*(*t*) the reward at time *t*, and **x**(*t*) and **w**(*t*) the state and weight vectors for the unit. A standard online TD learning rule was used with a fixed learning rate *α*, **w**(*t*) = **w**(*t *- 1) + *αδ*(*t*)**x**(*t *- 1), so each weight represented an expected future reward value. Similar to Fiorillo *et al*., we depict the prediction error *δ*(*t*) averaged over many trials, after the task has been learned. The representational asymmetry arises as negative values of *δ*(*t*) have been scaled by *d *= 1/6 prior to summation of the simulated PSTH, although learning proceeds according to unscaled errors. Finally, to account for the small positive responses at the time of the stimulus for *p*_*r *_= 0 and at the time of the (predicted) reward for *p*_*r *_= 1 seen in (a), we assumed a small (8%) chance that a predictive stimulus is misidentified. (c) DA response in *p*_*r *_= 0.5 trials, separated into rewarded (left) and unrewarded (right) trials. (d) TD Model of (c). (a,c) Reprinted with permission from [15]©2003 AAAS. Permission from AAAS is required for all other uses.

Figure 1a shows population histograms of extracellularly-recorded DA cell activity, for each *p*_*r*_. TD theory predicts that the phasic activation of the DA cells at the time of the visual stimuli should correspond to the *average *expected reward, and so should increase with *p*_*r*_. Figure 1a shows exactly this – indeed, across the population, the increase is quite linear. Morris *et al. *[16] report a similar result in an instrumental (trace) conditioning task also involving probabilistic reinforcement.

By contrast, at the time of potential reward delivery, TD theory predicts that *on average *there should be no activity, as, on average, there is no prediction error at that time. Of course, in the probabilistic reinforcement design (at least for *p*_*r *_≠ 0, 1) there is in fact a prediction error at the time of delivery or non-delivery of reward on every single trial. On trials in which a reward is delivered, the prediction error should be positive (as the reward obtained is larger than the average reward expected). Conversely, on trials with no reward it should be negative (see Figure 1c). Crucially, under TD, the average of these differences, weighted by their probabilities of occurring, should be zero. If it is not zero, then this prediction error should act as a plasticity signal, changing the predictions until there is no prediction error. At variance with this expectation, the data in Figure 1a which is averaged over both rewarded and unrewarded trials, show that there is in fact positive mean activity at this time. This is also evident in the data of Morris *et al. *[16] (see Figure 3c). The positive DA responses show no signs of disappearing even with substantial training (over the course of months).

Worse than this for the TD model, and indeed the focus of Fiorillo *et al. *[15], is the apparent *ramping *of DA activity towards the expected time of the reward. As the magnitude of the ramp is greatest for *p*_*r *_= 0.5, Fiorillo *et al. *suggested that it reports the *uncertainty *in reward delivery, *rather than *a prediction error, and speculated that this signal could explain the apparently appetitive properties of uncertainty (as seen in gambling).

Both the ramping activity and the activity at the expected time of reward pose critical challenges to the TD theory. TD learning operates by arranging for DA activity at one time in a trial to be *predicted away *by cues available earlier in that trial. Thus, it is not clear how any seemingly predictable activity, be it that at the time of the reward or in the ramp before, can persist without being predicted away by the onset of the visual stimulus. After all, the *p*_*r*_-dependent activity in response to the stimulus confirms its status as a valid predictor. Furthermore, a key aspect of TD [17], is that it couples prediction to action choice by using the *value *of a state as an indication of the future rewards available from that state, and therefore its attractiveness as a target for action. From this perspective, since the ramping activity is explicitly not predicted by the earlier cue, it cannot influence early actions, such as the decision to gamble. For instance, consider a competition between two actions: one eventually leading to a state with a deterministic reward and therefore no ramp, and the other leading to a state followed by a probabilistic reward with the same mean, and a ramp. Since the ramp does not affect the activity at the time of the conditioned stimulus, it cannot be used to evaluate or favour the second action (gambling) over the first, despite the extra uncertainty.

We suggest the alternative hypothesis that both these anomalous firing patterns result directly from the constraints implied by the low baseline rate of activity of DA neurons (2–4 Hz) on the coding of the *signed *prediction error. As noted by Fiorillo *et al. *[15], positive prediction errors are represented by firing rates of ~270% *above *baseline, while negative errors are represented by a decrease of only ~55% *below *baseline (see also [14,18]). This asymmetry is a straightforward consequence of the coding of a signed quantity by firing which has a low baseline, though, obviously, can only be positive. Firing rates above baseline can encode positive prediction errors by using a large dynamic range, however, below baseline firing rates can only go down to zero, imposing a restriction on coding of negative prediction errors.

Consequently, one has to be careful interpreting the sums (or averages) of peri-stimulus-time-histograms (PSTHs) of activity over different trials, as was done in Figure 1a. The asymmetrically coded positive and negative error signals at the time of the receipt or non-receipt of reward should indeed *not *sum up to zero, even if they represent correct TD prediction errors. When summed, the low firing representing the negative errors in the unrewarded trials will not "cancel out" the rapid firing encoding positive errors in the rewarded trials, and, overall, the average will show a positive response. In the brain, of course, as responses are not averaged over (rewarded and unrewarded) trials, but over neurons within a trial, this need not pose a problem.

This explains the persistent positive activity (on average) at the time of delivery or non-delivery of the reward. But what about the ramp prior to this time? At least in certain neural representations of the time between stimulus and reward, when trials are averaged, this same asymmetry leads TD to result exactly in a ramping of activity toward the time of the reward. The TD learning mechanism has the effect of propagating, on a trial-by-trial basis, prediction errors that arise at one time in a trial (such as at the time of the reward) towards potential predictors (such as the CS) that arise at earlier times within each trial. Under the asymmetric representation of positive and negative prediction errors that we have just discussed, averaging these propagating errors over multiple trials (as in Figure 1a) will lead to positive means for epochs within a trial before a reward. The precise shape of the resulting ramp of activity depends on the way stimuli are represented over time, as well as on the speed of learning, as will be discussed below.

**Figure 2:**
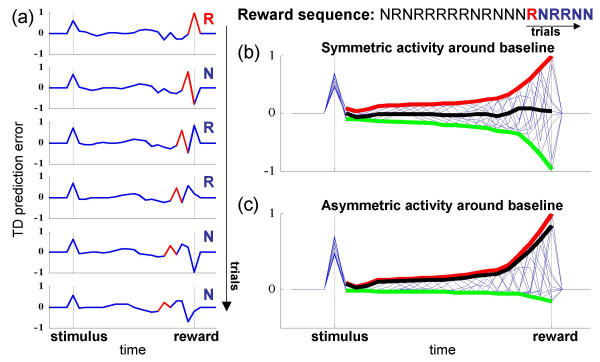
**Backpropagation of prediction errors explains ramping activity**. (a) The TD prediction error across each of six consecutive trials (top to bottom) from the simulation in Figure 1b, with *p*_*r *_= 0.5. Highlighted in red is the error at the time of the reward in the first of the trials, and its gradual back-propagation towards the time of the stimulus in subsequent trials. Block letters indicate the outcome of each specific trial (R = rewarded; N = not rewarded). The sequence of rewards preceding these trials is given on the top right. (b) The TD error from these six trials, and four more following them, superimposed. The red and green lines illustrate the envelope of the errors in these trials. Summing over these trials results in no above-baseline activity on average (black line), as positive and negative errors occur at random 50% of the time, and so cancel each other. (c) However, when the prediction errors are asymmetrically represented above and below the baseline firing rate (here negative errors were asymmetrically scaled by *d *= 1/6 to simulate the asymmetric encoding of prediction errors by DA neurons), an average ramping of activity emerges when averaging over trials, as is illustrated by the black line. All simulation parameters are the same as in Figure 1b,d.

Figure 2 illustrates this view of the provenance of the ramping activity. Here, a tapped delay-line representation of time since the stimulus is used. For this, each unit ('neuron') becomes active (i.e., assumes the value 1) at a certain lag after the stimulus has been presented, so that every timestep after the stimulus onset is consistently represented by the firing of one unit. Learning is based on the (dopaminergically-reported) TD error, formalized as *δ*(*t*) = *r*(*t*) + *V*(*t*) - *V*(*t *- 1), with *V*(*t*) the weighted input from the active unit at time *t*, and *r*(*t*) the reward obtained at time *t*. Updating the weights of the units according to the standard TD update rule with a fixed learning rate, allows *V*(*t*) to, on average, represent the expected future rewards (see Figure 1 caption). As each subsequent timestep is separately represented, TD prediction errors can arise at any time within the trial. Figure 2a shows these errors in six consecutive simulated trials in which *p*_*r *_= 0.5. In every trial, a new positive or negative error arises at the time of the reward, consequent on receipt or non-receipt of the reward, and step-by-step the errors from previous trials propagate back to the time of the stimulus, through the constant updating of the weights (*eg. *the error highlighted in red). When averaging (or, as in PSTHs, summing) over trials, these errors cancel each other on average, resulting in an overall flat histogram in the interval after the stimulus onset, and leading up to the time of the reward (black line in Figure 2b, summed over the 10 trials shown in thin blue). However, when summed after *asymmetric scaling *of the negative errors by a factor of *d *= 1/6 (which simulates the asymmetric coding of positive and negative prediction errors by DA neurons), a positive ramp of activity ensues, as illustrated by the black line in Figure 2c. Note that this rescaling is only a *representational *issue, resulting from the constraints of encoding a negative value about a low baseline firing rate, and should not affect the learning of the weights, so as not to learn wrong values (see discussion). However, as PSTHs are directly sums of neuronal spikes, this representational issue bears on the resulting histogram.

Figures 1b,d show the ramp arising from this combination of asymmetric coding and inter-trial averaging, for comparison with the experimental data. Figure 1b shows the PSTH computed from our simulated data by averaging over the asymmetrically-represented *δ*(*t*) signal in ~50 trials for each stimulus type. Figure 1d shows the results for the *p*_*r *_= 0.5 case, divided into rewarded and unrewarded trials for comparison with Figure 1c. The simulated results resemble the experimental data closely in that they replicate the net positive response to the uncertain rewards, as well as the ramping effect, which is highest in the *p*_*r *_= 0.5 case.

It is simple to derive the average response at the time of the reward (*t = N*) in trial *T*, *i.e., *the average TD error *δ*_*T*_(*N*), from the TD learning rule with the simplified tapped delay-line time representation and a fixed learning rate *α*. The value at the next to last timestep in a trial, as a function of trial number (with initial values taken to be zero), is



where *r*(*t*) is the reward at the end of trial *t*. The error signal at the last timestep of trial *T *is simply the difference between the obtained reward *r*(*T*), and the value predicting that reward *V*_*T *- 1 _(*N *- 1). This error is positive with probability *p*_*r*_, and negative with probability (1 - *p*_*r*_). Scaling the negative errors by a factor of *d *∈ (0, 1], we thus get



For symmetric coding of positive and negative errors (*d *= 1), the average response is 0. For asymmetric coding (0 <*d *< 1), the average response is indeed proportional to the variance of the rewards, and thus maximal at *p*_*r *_= 0.5. However, *δ*_*T *_is positive, and concomitantly, the ramps are positive, and in this particular setting, are related to uncertainty, *because of*, rather than *instead of*, the coding of *δ*(*t*).

Indeed, there is a key difference between the uncertainty and TD accounts of the ramping activity. According to the former, the ramping is a within-trial phenomena, coding uncertainty in reward; by contrast, the latter suggests that ramps arise only through averaging across multiple trials. Within a trial, when averaging over simultaneously recorded neurons rather than trials, the traces should not show a smooth ramp, but intermittent positive and negative activity corresponding to back-propagating prediction errors from the immediately previous trials (as in Figure 2a).

## Trace conditioning: a test case

**Figure 3:**
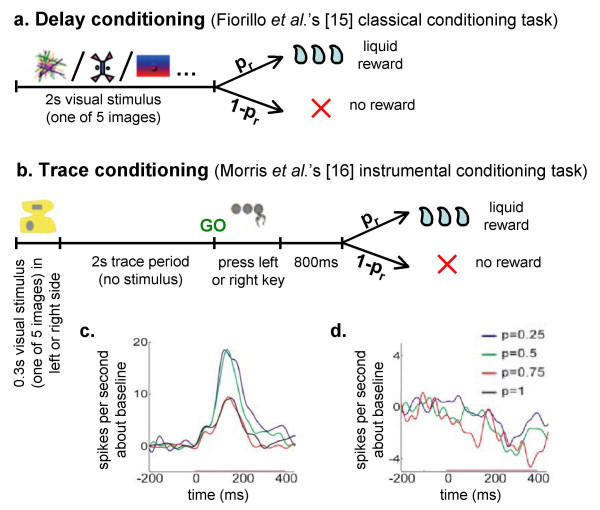
**Trace conditioning with probabilistic rewards**. (a) An illustration of one trial of the delay conditioning task of Fiorillo *et al. *[15]. A trial consists of a 2-second visual stimulus, the offset of which coincides with the delivery of the juice reward, if such a reward is programmed according to the probability associated with the visual cue. In unrewarded trials the stimulus terminated without a reward. In both cases an inter-trial interval of 9 seconds on average separates trials. (b) An illustration of one trial of the trace conditioning task of Morris *et al. *[16]. The crucial difference is that there is now a substantial temporal delay between the offset of the stimulus and the onset of the reward (the "trace" period), and no external stimulus indicates the expected time of reward. This confers additional uncertainty as precise timing of the predicted reward must be internally resolved, especially in unrewarded trials. In this task, as in [15], one of several visual stimuli (not shown) was presented in each trial, and each stimulus was associated with a probability of reward. Here, also, the monkey was requested to perform an instrumental response (pressing the key corresponding to the side in which the stimulus was presented), the failure of which terminated the trial without a reward. Trials were separated by variable inter-trial intervals. (c,d) DA firing rate (smoothed) relative to baseline, around the expected time of the reward, in rewarded trials (c) and in unrewarded trials (d). (c,d) Reprinted from [16] ©2004 with permission from Elsevier. The traces imply an overall positive response at the expected time of the reward, but with a very small, or no ramp preceding this. Similar results were obtained in a classical conditioning task briefly described in [15], which employed a trace conditioning procedure, confirming that the trace period, and not the instrumental nature of the task depicted in (b) was the crucial difference from (a).

An important test case for our interpretation arises in a variant of Fiorillo *et al.'s *[15] task, as well as in the analogous instrumental task of Morris *et al. *[16], both involving trace conditioning. In contrast to delay conditioning (Figure 3a) in which the reward coincides with the offset of the predictive stimulus, here there is a substantial gap between the offset of the predictive stimulus and the delivery of the reward (Figure 3b). Clearly, in this case, uncertainty about the reward could only get larger, owing to noise in timing the interval between stimulus and reward [19], so under the uncertainty account, there should be comparable or even larger ramps. However, the experimental results show the ramping activity to be *smaller*, or even negligible (Figure 3c;d). Note, though, that the magnitude of the trial-average activity at the expected time of reward is maintained, pointing to a dissociation between the height of the ramp and the amount of positive activity at the expected time of reward.

**Figure 4:**
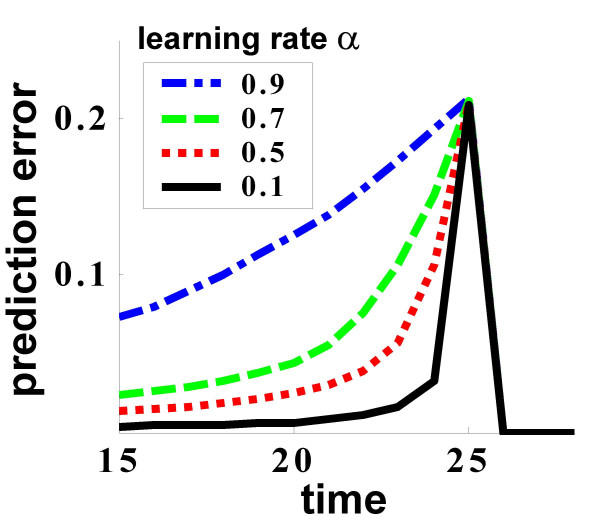
**Dependence of the ramp on learning rate. **The shape of the ramp, but not the height of its peak, is dependent on the learning rate. The graph shows simulated activity for the case of *p*_*r *_= 0.5 near the time of the expected reward, for different learning rates, averaged over both rewarded and unrewarded trials. According to TD learning with persistent asymmetrically coded prediction errors, averaging over activity in rewarded and unrewarded trials results in a ramp up to the time of reward. The height of the peak of the ramp is determined by the ratio of rewarded and unrewarded trials, however, the breadth of the ramp is determined by the rate of back-propagation of these error signals from the time of the (expected) reward to the time of the predictive stimulus. A higher learning rate results in a larger fraction of the error propagating back, and thus a higher ramp. With lower learning rates, the ramp becomes negligible, although the positive activity (on average) at the time of reward is still maintained. Note that although the learning rate used in the simulations depicted in Figure 1b,d was 0.8, this should not be taken as the literal synaptic learning rate of the neural substrate, given our schematic representation of the stimulus. In a more realistic representation in which a population of neurons is active at every timestep, a much lower learning rate would produce similar results.

The TD model of DA readily explains these puzzling data. As shown in Figure 4, the shape of the ramp, though not the height of its peak, is affected by the learning rate. The size of the back-propagating prediction errors is determined, in part, by the learning rate, as these errors arise as part of the online learning of new predictions. Indeed, there is a continuous updating of predictions such that after a rewarded trial, there is a higher expectation of reward (and thus the next reward incurs a smaller prediction error), and conversely after a non-rewarded trial [18] (see Figure 2a). This updating of predictions is directly related to the learning rate – the higher the learning rate, the larger the update of predictions according to the current prediction error, and the larger the fraction of the prediction error which is propagated back. In this way, with higher learning rates, the difference in expectations after a rewarded versus an unrewarded trial will be larger, and thus the prediction errors when the next reward is or is not available will be larger – hence the larger and more gradual ramp.

Indeed, compared to delay conditioning, trace conditioning is notoriously slow, suggesting that the learning rate is low, and thus that there should be a lower ramp, in accord with the experimental results. A direct examination of the learning rate in the data of Morris *et al. *[16], whose task required excessive training as it was not only a trace conditioning one but also involved an instrumental action, confirmed it indeed to be very low (Genela Morris – personal communication, 2004).

## Discussion

The differential coding of positive and negative values by DA neurons is evident in all the studies of the phasic DA signal, and can be regarded as an inevitable consequence of the low baseline activity of these neurons. Indeed, the latter has directly inspired suggestions that an opponent neurotransmitter, putatively serotonin, be involved in representing and therefore learning the negative prediction errors [20], so that they also have full quarter. Here, however, we have confined ourselves to consideration of the effects of asymmetry on the trial-average analysis of the dopamine activity, and have shown that ramping DA activity, as well as an average positive response at the time of reward, result directly from the asymmetric coding of prediction errors.

Apart from a clearer view of the error signal, the most important consequence of the new interpretation is that the ramps can be seen as a signature of a TD phenomenon that has hitherto been extremely elusive. This is the progressive back-propagation of the error signal represented by DA activity, from the time of reward to the time of the predictor (Figure 2a). Most previous studies of dopaminergic activity have used *p*_*r *_= 1, so making this back-propagation at best a transitory phenomenon apparent only at the beginning of training (when, typically, recordings have not yet begun), and potentially hard to discern in slow-firing DA neurons. Further, as mentioned, the back-propagation depends on the way that the time between the predictive stimulus and the reward is represented – it is present for a tapped delay-line representation as in [6], but not for representations which span the entire delay, such as in [21]. Note that the shape of the ramp also depends on the use of eligibility traces and the so-called TD(*λ*) learning rule (simulation not shown), which provide an additional mechanism for bridging time between events during learning. Unfortunately, as the forms of the ramps in the data are rather variable (figure 1) and noisy, they can not provide strong constraints on the precise TD mechanism used by the brain.

More recent studies involving persistent prediction errors also show activity suggestive of back-propagation, notably Figure 4 of [13]. In this study, prediction errors resulted from periodic changes in the task, and DA recordings were made from the onset of training, thus back-propagation-like activity is directly apparent, although this activity was not quantified.

We expect the ramps to persist throughout training only if the learning rate does not decrease to zero as learning progresses. Pearce & Hall's [22] theory of the control of learning by uncertainty suggests exactly this persistence of learning – and there is evidence from partial reinforcement schedules that the learning rate may be higher when there is more uncertainty associated with the reward. Indeed, from a 'rational' statistical point of view, learning should persist when there is substantial uncertainty about the relationship between predictors and outcomes, as can arise from the ever-present possibility of a change in the predictive relationships. This form of persistent uncertainty, together with uncertainty due to initial ignorance regarding the task, have been used to formalize Pearce & Hall's theory of the way that uncertainty drives learning [23]. Thus, our claim that uncertainty may not be directly represented by the ramps, should certainly not be taken to mean that its representation and manipulation is not important. To the contrary, we have suggested that uncertainty influences cortical inference and learning through other neuromodulatory systems [24], and that it also may determine aspects of the selection of actions [25].

Various other features of the asymmetry should be noted. Most critical is the effect of the asymmetry on DA-dependent learning [26], if the below baseline DA activity is responsible by itself for decreasing predictions which are too high. In order to ensure that the learned predictions remain correct, we would have to assume that the asymmetric representation does not affect learning, *i.e., *that a mechanism such as different scaling for potentiation and depression of the synaptic strengths compensates for the asymmetric error signal. Of course, this would be rendered moot if an opponent neurotransmitter is involved in learning from negative prediction errors. This issue is complicated by the suggestion of Bayer [14] that DA firing rates are actually similar for all prediction errors below some negative threshold, perhaps due to the floor effect of the low firing rate. Such lossy encoding does not affect the qualitative picture of the effects of inter-trial averaging on the emergence of ramps, but does reinforce the need for an opponent signal for the necessarily symmetric learning.

Finally, the most direct test of our interpretation would be a comparison of intra- and inter-trial averaging of the DA signal. It would be important to do this in a temporally sophisticated manner, to avoid problems of averaging non-stationary signals. In order to overcome the noise in the neural firing, and determine whether indeed there was a gradual ramp within a trial, or, as we would predict – intermittent positive and negative prediction errors, it would be necessary to average over many neurons recorded simultaneously within one trial, and furthermore neurons associated with similar learning rates. Alternatively, single neuron traces could be regressed against the backpropagation response predicted by their preceding trials and TD learning. A comparison of the amount of variability explained by such a model, compared to that from a regression against a monotonic ramp of activity, could point to the most fitting model. A less straightforward, but more testable prediction is that the shape of the ramp should depend on the learning rate. Learning rates can be assessed from the response to the probabilistic rewards, independent of the shape of the ramp (Nakahara *et al. *[18] showed in such a way, that in their partial reinforcement trace conditioning task, the learning rate was 0.3), and potentially manipulated by varying the amount of training or the frequency with which task contingencies are changed and relearned. Indeed, quantifying the existence and shape of a ramp in Nakahara *et al.'s *recorded DA activity, could well shed light on the current proposal.

## Competing interests

The author(s) declare that they have no competing interests.

## Authors' contributions

YN, MD and PD jointly conceived and executed this study, and helped draft the manuscript. All authors read and approved the final manuscript.
